# Telomere length, proviral load and neurologic impairment in HTLV-1-and HTLV-2-infected humans

**DOI:** 10.1186/1742-4690-11-S1-O15

**Published:** 2014-01-07

**Authors:** Benjamin Usadi, Roberta Bruhn, Jue Lin, Tzong-Hae Lee, Elizabeth Blackburn, Edward L Murphy

**Affiliations:** 1 Blood Systems Research Institute, San Francisco, CA, USA; 2 University of California, Berkeley, Berkeley, CA, USA; 3 University of California San Francisco, San Francisco, CA, USA

## Background

Telomeres shorten with aging and short or damaged telomeres have been implicated in degenerative conditions. We hypothesized that telomere length might be altered in chronic HTLV-1 and -2 infection and could be a marker of HTLV-associated disease and viral dynamics.

## Methods

45 HTLV-1, 45 HTLV-2, and 45 seronegative subjects were selected from the larger HTLV Outcomes Study (HOST) cohort, and stratum-matched on age, sex and race/ethnicity. The telomere-to-single copy gene (T/S) ratio and HTLV-1 and -2 proviral load were measured using real-time PCR on the same PBMC samples. Unpaired t-tests, linear regression and logistic regression were used to test associations.

## Results

Ln T/S ratio was inversely associated with age among seronegatives (p=.006) but HTLV-1 and -2 subjects did not show an inverse age association. There was no difference in mean T/S ratio between HTLV-1 (1.02), HTLV-2 (1.03) and matched seronegative (0.99) subjects. In HTLV-1 subjects, there was a borderline inverse association (p=0.07) between T/S ratio and log10 proviral load which did not persist after multivariate adjustment (p=0.17). Among HTLV-2 subjects only, Ln T/S ratio was significantly associated (p=0.026) with increased odds of vibration-sensation impairment.

## Conclusions

We found no evidence for an overall difference in telomere length between HTLV cases and controls but there was a weak association between HTLV-1 proviral load and telomere length. The association between telomere length and impaired vibration sense in the HTLV-2-positive group is intriguing, and suggests avenues for future investigation of previously described neuropathy in that group.

